# Placenta and Umbilical Cord Cause in Antepartum Deaths

**DOI:** 10.7759/cureus.3556

**Published:** 2018-11-07

**Authors:** Xenofon Mantakas, Ioannis Dalivigkas, Leon Aravantinos, Nikos Goutas, Christina Goudeli, Nikos Vlahos

**Affiliations:** 1 Pathology, Aretaieio Hospital, Athens, GRC; 2 Obstetrics and Gynecology, Attikon Hospital, Athens, GRC; 3 Obstetrics and Gynecology, Aretaieio Hospital, Athens, GRC; 4 Pathology, Medical School of Athens, Athens, GRC; 5 Obstetrics and Gynecology, “Saint Savvas” Cancer Hospital, Athens, GRC

**Keywords:** stillbirth, antepartum death, placenta, umbilical cord, second trimester

## Abstract

Stillbirth is a sudden and painful event for parents and obstetrical specialists as well. It is, therefore, of greatest importance to be able to give answers for the cause in order to plan a subsequent pregnancy. The aim of this retrospective study is to estimate the placental and umbilical cord cause of intrauterine death in relation to different gestational ages. The study took place on the Medical Birth Registry of Aretaieio Hospital, National and Kapodistrian University, Athens, Greece. We include a total of 19,283 pregnancies from 1998 to 2012. In this study period, 431 embryonic deaths occurred. The clinical history was documented on admission at delivery. Conditions thought to be associated with the intrauterine fetal death were recorded. Gestational age was calculated from the last menstrual period as well as with the three-trimester system. The autopsy, placenta and umbilical cord examination were performed by the same laboratory of pathology in Aretaieio University Hospital. We found that the majority of stillbirths occurred in the second trimester. We examined placenta and umbilical cord in all cases. The most frequent histologic abnormalities were those indicated placental vascular insufficiency. As far as the umbilical cord is concerned we found that the inflammatory disorder was the most common in antepartum deaths. A single umbilical artery was significantly related to gestational diabetes and congenital embryonic anomalies. Finally, our results showed steady declines in antepartum deaths during 1998-2012. As a result, we reached the conclusion that in order to reduce the fetal death rate, we have to insist on the autopsy of the placenta and umbilical cord in order to gain the appropriate information in counseling the parents.

## Introduction

The intrauterine fetal or antepartum death of second and third trimester, especially in pregnancies without any risk factor, is often unexpected and unexplained [1-3]. As per the definition by World Health Organization (WHO), stillbirth or fetal death is death prior to the complete expulsion or extraction from its mother of a product of conception, irrespective of the duration of pregnancy; the death is indicated by the fact that after such separation the fetus does not breathe or show any other sign of life, such as beating of the heart, pulsation of the umbilical cord or definite movement of voluntary muscles [4]. In developed countries, where interventions have largely eliminated excess early neonatal mortality, over six out of 10 perinatal deaths are stillbirths [5]. There are many references to unexpected fetal deaths related to placental dysfunction or to umbilical cord cause [6-8]. Placental bed pathology involves inadequate spiral artery pathology or both leading to maternal vascular under perfusion [6, 9, 10]. It involves as well abnormalities in the parenchyma that are reflected in the weight of the placenta, placental hypoplasia and many other dysfunctions like placental infection and abnormal development of placenta villous [6,10,11]. There are currently at least 32 classification systems of stillbirth, many of which have been developed for different purposes [12,13]. They have different categories of classifying causes. In the new ones TULIP-Relevant Condition at Death (RECODE-UK) placenta and umbilical cord pathology consist the most common cause of fetal death [9]. Many other classification systems have no mention on the placenta and therefore the largest group of fetal death remains nonspecific [6, 7, 14]. Antepartum death can be divided by gestational age into early (20-28 weeks) or late deaths (more than 28 weeks) or into the second and third trimester. At the same time, a conventional autopsy is considered the diagnostic gold standard, because it can confirm or augment antemortem findings, fulfill the need for placental information, assist with future planning as well as provide answers about what had happened [3, 15, 16]. The aim of this retrospective study is to estimate the placental cause of intrauterine death in relation to different gestational ages. It is important to note that no other study with this time duration, parameters, and a large population has ever been released in Greece [17, 18].

## Materials and methods

The purpose was to study the dysfunctions of the placenta and umbilical cord in all antepartum deaths after the second trimester in Aretaieio University Hospital of Athens from 1998 to 2012. The study was a population-based registry study. We excluded stillbirths less than second trimester (20 weeks) of gestation and therapeutic abortions-terminated pregnancies. Any additional births that lacked information on the placenta and umbilical cord autopsy were also excluded. As a result, we included 19,283 births. Gestational age was calculated from the last menstrual period as well as with the three-trimester system. In few cases of 1990s that the gestational age was not provided, we used the Crown-Rump Length (CRL) to calculate the gestational week. It is however unlikely that change in the estimation of gestational length has significantly biased our results because gestational age was defined mainly as three trimesters. Among the cases, we included 49 multiple/twin pregnancies (47 twins and two triplets) of at least one death of the embryo. The clinical history was documented on admission at delivery. Conditions thought to be associated with the intrauterine fetal death were recorded. The autopsy and placenta and umbilical cord examination were performed by the same laboratory in Aretaieio University Hospital. Quantitative variables were expressed as mean values (SD), while qualitative variables were expressed as absolute and relative frequencies. For the comparisons of proportions, chi-square and Fisher’s exact tests were computed. Analysis of variance (ANOVA) was used for the comparison of mean values. Rates of embryonic death per 1000 ongoing pregnancies were calculated in the total sample and by year of delivery. Relative risks of embryonic death and their 95% confidence intervals were calculated for each time period, with 1998-2002 as the reference period. Statistical significance was set at p < 0.05 and analyses were conducted using SPSS statistical software version 22.0 (IBM Corp., Armonk, NY).

## Results

A total of 19,283 pregnancies in Aretaieio University Hospital of Athens from 1998 to 2012 were included in the study. In the study period, 431 embryonic deaths (23.9%) occurred. Characteristics of the embryonic deaths, pregnancies and mother's demographics are presented in Table 1.

**Table 1:** Embryonic deaths, maternal and pregnancies characteristics.

	N (%)
Mother's age, mean (SD)	31.7 (5.3)
Gender	
Males	239 (51.8)
Females	222 (48.2)
Gestational week, mean (SD)	21.8 (6.2)
Gestational week	
<20th	177 (38.4)
20th-27th	212 (46.0)
>27th	72 (15.6)
Trimester	
2nd	376 (81.6)
3rd	85 (18.4)
Mixed	124 (26.9)
Nonspecific cause	13 (2.8)
Congenital embryonic anomalies	14 (3.0)
If yes, define	
Polycystic kidney dysplasia	4 (0.9)
Congenital heart disease	6 (1.3)
Tail tracheal aggression syndrome	1 (0.2)
Fallot tetralogy	3 (0.7)
Embryonic vascular disorders	7 (1.5)
If yes, define	
Embryonic vascular thrombopathy	1 (0.2)
Intrauterine transfusion syndrome	6 (1.3)
Pregnancy diseases	18 (3.9)
If yes, define	
Cytomegalovirus (CMV)	1 (0.2)
Diabetes	16 (3.5)
Toxoplasma	1 (0.2)
In vitro fertilization (IVF)	38 (8.2)

The majority of the embryonic deaths occurred between 20th and 27th week of gestation (46.0%). The mean age of the mother was 31.7 years (SD = 5.3). Among the deaths, 239 were males (51.8%). Most losses occurred in the second trimester (81.6%). Congenital embryonic anomalies were present in 3% of the cases and embryonic vascular disorders in 1.5% of the cases. Diabetes was recorded in 3.5% of the pregnancies. In 38 cases (8.2%) in vitro fertilization (IVF) was performed as it can be seen in Table 1. Table 2 shows the results of placenta examination associated with embryonic deaths. The mean placental weight was 214.7 gr (SD = 119.8) and the mean placenta diameter was 13.1 cm (SD = 4.0). Vascular disorders were recorded in 38.6% of the samples and inflammatory disorders in 35.6% of the samples. Functional-structural disorders were present in 28.0% of the placentas and abruption in 13.7% of them.

**Table 2:** Results from placenta examination associated with embryonic deaths.

	N (%)
Placenta weight (gr), mean (SD)	214.7 (119.8)
Placenta diameter (cm), mean (SD)	13.1 (4.0)
Vascular disorders	178 (38.6)
If yes, define	
Occlusion	75 (16.3)
Occlusion-intravillus haematomas	46 (10.0)
Occlusion-intervillous haematomas-thrombi	57 (12.4)
Inflammatory disorders	164 (35.6)
If yes, define	
Infection of villus and necrosis after certain infection	2 (0.4)
Infection of villus	8 (1.7)
Acute-in part necrotic chorioamnionitis	57 (12.4)
Acute chorioamnionitis	93 (20.2)
Acute chorioamnionitis and infection of placenta	2 (0.4)
Functional-structural disorders	129 (28.0)
If yes, define	
Villi immaturity	52 (11.3)
Fibrin deposition	54 (11.7)
Necrosis	3 (0.7)
Villi necrosis	5 (1.1)
Intrauterine transfusion syndrome	1 (0.2)
Hydrophilic	13 (2.8)
Placenta abruption	63 (13.7)
If yes, define	
Backplate haematomas	63 (13.7)

**Figure 1:**
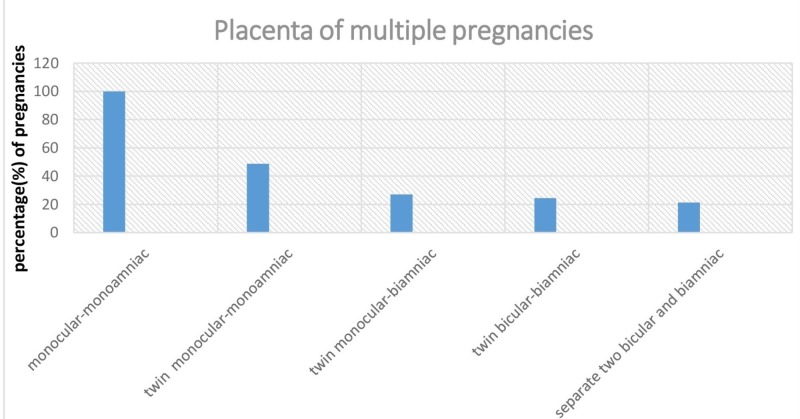
Placenta and multiple pregnancies of at least one stillbirth.

As it can be seen in Figure 1 the most common placenta of multiple pregnancies of at least one stillbirth was a monocular one.

Results from placenta and umbilical cord examination according to the time period and gestational week are presented in Table 3.

**Table 3:** Results from placenta and umbilical cord examination according to time period and gestational week. ^+ ^Pearson's chi-square test; ^++ ^Fisher’s exact test; ^‡ ^ANOVA

	Year			P	Gestational week			P
	1998-2002	2003-2007	2008-2012		<20th	20th-27th	>27th	
	N (%)	N (%)	N (%)		N (%)	N (%)	N (%)	
Placenta								
Vascular disorders	66 (36.9)	53 (39.6)	59 (39.9)	0.828^+^	59 (33.3)	91 (42.9)	28 (38.9)	0.154^+^
Inflammatory disorders	58 (32.4)	56 (41.8)	50 (33.8)	0.197^+^	73 (41.2)	73 (34.4)	18 (25.0)	0.047^+^
Functional-structural disorders	45 (25.1)	36 (26.9)	48 (32.4)	0.324^+^	56 (31.6)	53 (25.0)	20 (27.8)	0.348^+^
Placenta abruption	22 (12.3)	22 (16.4)	19 (12.8)	0.540^+^	25 (14.1)	31 (14.6)	7 (9.7)	0.564^+^
Weight (gr), mean (SD)	214.9 (113.1)	211.4 (131.8)	217.4 (117.1)	0.918^‡^	127.9 (61.4)	221.2 (56.8)	406.8 (134.2)	<0.001^‡^
Diameter (cm), mean (SD)	13.8 (4.6)	13 (3.6)	12.4 (3.5)	0.005^‡^	10.1 (2.9)	13.9 (2.4)	18.3 (3.9)	<0.001^‡^
Umbilical cord								
Vascular disorders	10 (5.6)	8 (6.0)	11 (7.4)	0.781^+^	7 (4.0)	14 (6.6)	8 (11.1)	0.107^+^
Inflammatory disorders	27 (15.1)	18 (13.5)	19 (12.8)	0.834^+^	27 (15.3)	29 (13.7)	8 (11.1)	0.677^+^
Tourniquet	7 (3.9)	9 (6.8)	6 (4.1)	0.445^+^	2 (1.1)	5 (2.4)	15 (20.8)	<0.001^+^
Single umbilical artery	9 (5.0)	8 (6.0)	8 (5.4)	0.930^+^	12 (6.8)	11 (5.2)	2 (2.8)	0.434^+^
Umbilical cord adhesion								
Eccentric	156 (87.2)	111 (83.5)	120 (81.1)	0.495^+^	156 (88.6)	173 (81.6)	58 (80.6)	0.291^+^
Low	16 (8.9)	17 (12.8)	23 (15.5)		14 (8.0)	31 (14.6)	11 (15.3)	
Hymenus	7 (3.9)	5 (3.8)	5 (3.4)		6 (3.4)	8 (3.8)	3 (4.2)	
Length, mean (SD)	22 (10.7)	23.8 (21.1)	17.3 (10.5)	<0.001^‡^	16.1 (17.3)	21 (7.8)	33.2 (16.2)	<0.001^‡^
Diameter, mean (SD)	0.91 (0.30)	0.81 (0.30)	0.77 (0.30)	<0.001^‡^	0.68 (0.30)	0.87 (0.30)	1.15 (0.30)	<0.001^‡^
Mixed	47 (26.3)	37 (27.6)	40 (27.0)	0.964^+^	36 (20.3)	68 (32.1)	20 (27.8)	0.034^+^
Nonspecific cause	6 (3.4)	3 (2.2)	4 (2.7)	0.938^++^	4 (2.3)	6 (2.8)	3 (4.2)	0.644^++^
Congenital embryonic anomalies	7 (3.9)	6 (4.5)	1 (0.7)	0.099^++^	5 (2.8)	7 (3.3)	2 (2.8)	0.954^+^
Embryonic vascular disorders	0 (0.0)	3 (2.2)	4 (2.7)	0.057^++^	0 (0.0)	5 (2.4)	2 (2.8)	0.065^++^
Pregnancy diseases	8 (4.5)	6 (4.5)	4 (2.7)	0.657^+^	2 (1.1)	11 (5.2)	5 (6.9)	0.042^+^
In vitro Fertilization (IVF)	6 (3.4)	10 (7.5)	22 (14.9)	0.001^+^	14 (7.9)	20 (9.4)	4 (5.6)	0.574^+^

Inflammatory disorders of the placenta decreased significantly as the gestational week increases. As expected, both the weight and diameter of the placenta increased along with the increase of gestational week and the same result was found for the length and diameter of the umbilical cord. The diameter of the placenta and proportion of IVF also increased by the three time periods. Tourniquet was more frequent in greater gestational weeks. Also, the proportion of mixed and pregnancy diseases was lower in cases with gestational weeks fewer than 20. Table 4 shows results from umbilical cord examination.

**Table 4:** Results from umbilical cord examination associated with embryonic deaths.

	N (%)
Umbilical cord length (cm), mean (SD)	21.0 (14.7)
Umbilical cord diameter (cm), mean (SD)	0.84 (0.32)
Vascular disorders	29 (6.3)
If yes, define	29 (6.3)
Thrombosis	
Inflammatory disorders	64 (13.9)
If yes, define	
Degeneration-necrosis	1 (0.2)
Umbilical cord infection	21 (4.6)
Acute vasculitis	42 (9.1)
Tourniquet	22 (4.8)
Single umbilical artery	25 (5.4)
Umbilical cord adhesion	
Eccentric	387 (84.1)
Low	56 (12.2)
Hymenus	17 (3.7)

The mean umbilical cord length was 21 cm (SD = 14.7) and the mean umbilical cord diameter was 0.84 cm (SD = 0.32). Vascular disorders were recorded in 6.3% of the umbilical cord samples and inflammatory disorders in 13.9%. Tourniquet was found in 4.8% of the umbilical cords. The single umbilical artery was recorded in 5.4% of the samples. As it can be seen in Table 5, increased rates of gestational diabetes were found in cases where the single umbilical artery was found (28% vs. 2.1%, p < 0.001).

**Table 5:** Results from cases with single umbilical artery. ^ǁ ^Fisher's exact test; ^¶ ^Student's t-test

		Single Umbilical Artery				
		NO		YES		
		N	%	N	%	P
Pregnancy diabetes	no	426	97.9	18	72.0	<0.001ǁ
	yes	9	2.1	7	28.0	
Umbilical cord adhesion	eccentric	369	84.8	18	72.0	<0.001ǁ
	low	56	12.9	0	0.0	
	hymenus	10	2.3	7	28.0	
Placenta weight (gr), mean (SD)		216.4 (121.1)		184.0 (91.3)		0.198¶
Congenital embryonic abnormalities	no	429	98.6	17	68.0	<0.001ǁ
	yes	6	1.4	8	32.0	

Also, congenital embryonic anomalies were more frequent in cases where the single umbilical artery was found (32% vs. 1.4%, p < 0.001). Presence of vasculitis was more frequent in cases with thrombosis in umbilical cord (34.5% vs. 7.4%, p < 0.001) or chorioamnionitis (15.8% vs. 5.8%, p < 0.001), as it can be seen in Tables 6, 7.

**Table 6:** Results from cases with thrombosis. ^ǁ ^Fisher's exact test; ^¶ ^Student's t-test

		Thrombosis				P
		NO		YES		
		N	%	N	%	
Vasculitis	NO	399	92.6	19	65.5	<0.001ǁ
	YES	32	7.4	10	34.5	
Length of the umbilical cord, mean (SD)		20.7 (14.7)		25.2 (14.3)		0.114¶

**Table 7:** Results from cases with chorioamnionitis.

		Chorioamnionitis				P Pearson's x^2^ test
		NO		YES		
		N	%	N	%	
Vasculitis	no	290	94.2	128	84.2	<0.001
	yes	18	5.8	24	15.8	

The length of umbilical cord was significantly greater in cases with wrapping or with placenta abruption (p < 0.001), while the mean placental weight was greater in cases with gestational diabetes (p = 0.026), as it can be seen in Tables 8-10.

**Table 8:** Results from cases with single umbilical cord.

		Single umbilical cord		P Student's t-test
		Mean	SD	
Wrapping	No	19.6	13.0	<0.001
	Yes	49.0	18.3	

**Table 9:** Results from cases with placenta abruption. ^ǁ ^Pearson's x^2^ test; ^¶ ^Student's t-test

		Placenta abruption				P
		No		Yes		
		N	%	N	%	
Chorioamnionitis	No	259	65.1	50	79.4	0.025ǁ
	Yes	139	34.9	13	20.6	
Umbilical cord mean length		21.7 (15.5)		16.9 (6.3)		0.017¶

**Table 10:** Results from pregnancy diabetes.

	Pregnancy diabetes				P Student's t-test
	No		Yes		
	Mean	SD	Mean	SD	
Placenta weight (gr)	212.4	120.5	282.3	73.7	0.026

Furthermore, Table 11 shows that deposition of fibrin was significantly associated with the presence of occlusion-intervillous hematomas-thrombi (100% vs. 0.7%, p < 0.001) and vascular dysfunctions (100% vs. 30.5%, p < 0.001).

**Table 11:** Results from fibrin deposition.

		Fibrin deposition				P Pearson's x^2^ test
		No		Yes		
		N	%	N	%	
Occlusion-intravillus haematomas-thrombi	no	404	99.3	0	0.0	<0.001
	yes	3	0.7	54	100.0	
Vascular dysfunctions	no	283	69.5	0	0.0	<0.001
	yes	124	30.5	54	100.0	

**Figure 2:**
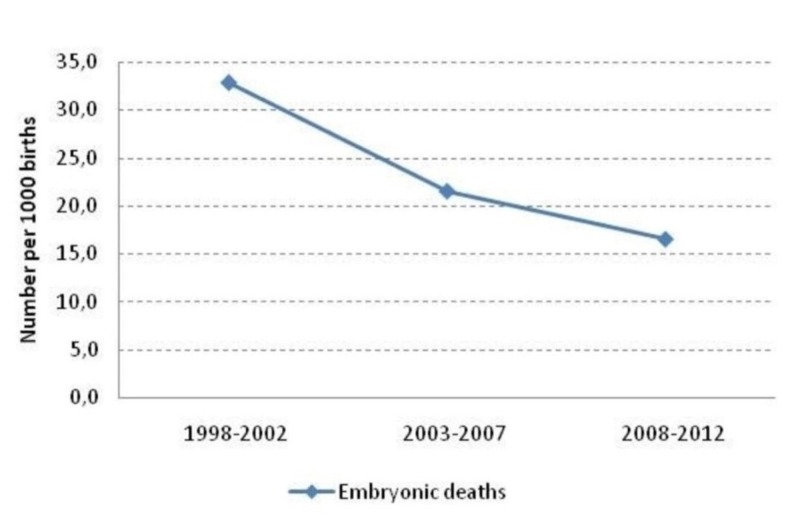
Embryonic death rates for the three time periods.

The embryonic death rate decreased by 49.7%, from 32.8 per 1000 births in the years 1998-2002 to 16.5 per 1000 births in the years 2008-2012 as it can be seen in Figure 2.

The relative risk of embryonic death in 2003-2007 was 0.66 (95% CI: 0.53–0.82) and significantly lower comparing births during 1998-2002, as it can be seen in Table 12. Also, the relative risk of embryonic death in 2008-2012 was 0.59 (95% CI: 0.48–0.74) and significantly lower comparing births during 1998-2002.

**Table 12:** Relative risks (RR) with 95% CI of embryonic death according to time period of delivery. ^* ^Relative Risk (95% Confidence Interval); ^‡ ^Indicates reference category

	Embryonic deaths	Births		
Year	N (%)	N (%)	Embryonic deaths (%)	RR (95% CI)*
1998-2002	179 (38.8)	5453 (28.3)	32.8	1.00^‡^
2003-2007	134 (29.1)	6227 (32.3)	21.5	0.66 (0.53 - 0.82)
2008-2012	148 (32.1)	7603 (39.4)	16.5	0.59 (0.48 - 0.74)
Total	461 (100.0)	19283 (100.0)	23.9	-

## Discussion

There are intensified demands on medical, political and epidemiological grounds for proper determination and classification of cause of antepartum deaths [19, 20]. Findings from this study confirm the necessity [16] of the microscopic examination of the placenta and umbilical cord for the purpose of parents counseling and prevention and for the comparison of health care nationally and internationally. Our findings agree with other published reports [16, 21, 22] that the majority of stillbirths occurred in the second trimester. This illustrates the vital role of the placenta [23] and umbilical cord for the optimal fetal development [23-25]. The slight predominance of stillbirths concerned male embryo and the mean maternal age was 31.7 years. Previous studies have shown that chromosomal abnormality rate was significantly higher in male miscarriages [26]. So, these results raise multiple questions for future research whether those chromosomal abnormalities could be related with the placenta or umbilical dysfunctions. The major frequent histologic abnormalities were those indicated by placental vascular insufficiency in accordance with previous studies [8, 10, 16]. The second significant placenta dysfunction was found in the inflammatory disorders. Other abnormalities such as large infractions, functional-structural disorders, and placenta abruption were also studied. They were not approved of sufficient significance to this study. In addition, we noticed that the majority of multiple pregnancies which had at least one stillbirth had a monocular placenta as it can be seen in Figure 1. The umbilical cord has been examined [27] and studied in placenta autopsies. In our study, we found that inflammatory disorder was the most common umbilical cord dysfunction in antepartum deaths. The vascular disorders were present only at 6.3%. Meanwhile, the eccentric adhesion of umbilical cord was found in most cases. This is rather due to the fact that the low and hymenus adhesion is rare than to the fact that the eccentric adhesion itself is importantly related to fetal deaths. In opposite to placenta cause, the wrapping of the umbilical cord was more frequent in the third-trimester (p < 0.001) which was expected since the length of the umbilical cord is increased in the third trimester [28]. We noticed that only 5.4% of the cases had a single umbilical artery as it can be seen in Table 4. Therefore, we suggest that neither the adhesion of umbilical cord nor the single umbilical artery had any special role in antepartum deaths. In agreement with other studies, we proved that the rates of gestational diabetes were significantly higher in cases with single umbilical artery (p < 0.001) as well as the congenital anomalies (p < 0.001) [29]. Finally, our results corroborate studies showing steady declines in antepartum deaths [30] but contrast with some studies that have shown stagnating or increasing stillbirth rates. The stillbirth declined at all gestational age which reflects changes in screening policies. Nowadays we have a better postnatal management of certain anomalies such as congenital heart defects. Early termination for lethal anomalies is also permitted and is not included in stillbirths. Therefore, there is a significant decline in stillbirths at all gestational age.

## Conclusions

The ability to predict and prevent stillbirth remains poor, as most stillbirths occur in women who are deemed to be at “low risk” of pregnancy complications. To reduce the fetal death rate, we need to gain more insight into the placenta causes and umbilical cord dysfunctions. This will give us the opportunity to reduce the morbidity and mortality in the setting of the placenta and umbilical cord. Obstetrical specialists should continue to carry a high index of suspicion in patients, especially in the second trimester. They have to insist on the autopsy of the placenta and umbilical cord as it remains the gold standard method in understanding the cause of an antepartum death. Through this procedure, they will gain the appropriate information for the best counseling of the parents and for planning any future childbearing.
